# Impact of the Coronavirus Pandemic (COVID-19) Lockdown on Mental Health and Well-Being in the United Arab Emirates

**DOI:** 10.3389/fpsyt.2021.633230

**Published:** 2021-03-16

**Authors:** Leila Cheikh Ismail, Maysm N. Mohamad, Mo'ath F. Bataineh, Abir Ajab, Amina M. Al-Marzouqi, Amjad H. Jarrar, Dima O. Abu Jamous, Habiba I. Ali, Haleama Al Sabbah, Hayder Hasan, Lily Stojanovska, Mona Hashim, Reyad R. Shaker Obaid, Sheima T. Saleh, Tareq M. Osaili, Ayesha S. Al Dhaheri

**Affiliations:** ^1^Department of Clinical Nutrition and Dietetics, College of Health Sciences, University of Sharjah, Sharjah, United Arab Emirates; ^2^Nuffield Department of Women's & Reproductive Health, University of Oxford, Oxford, United Kingdom; ^3^Research Institute of Medical and Health Sciences, University of Sharjah, Sharjah, United Arab Emirates; ^4^Department of Nutrition and Health, College of Medicine and Health Sciences, United Arab Emirates University, Al Ain, United Arab Emirates; ^5^Department of Sport Rehabilitation, Faculty of Physical Education and Sport Sciences, The Hashemite University, Zarqa, Jordan; ^6^Department of Health Services Administration, College of Health Sciences, Research Institute for Medical and Health Sciences, University of Sharjah, Sharjah, United Arab Emirates; ^7^College of Natural and Health Sciences, Zayed University, Dubai, United Arab Emirates; ^8^Institute for Health and Sport, Victoria University, Melbourne, VIC, Australia; ^9^Department of Nutrition and Food Technology, Faculty of Agriculture, Jordan University of Science and Technology, Irbid, Jordan

**Keywords:** psychological impact, mental health, COVID-19, United Arab Emirates, well-being

## Abstract

United Arab Emirates (UAE) has taken unprecedented precautionary measures including complete lockdowns against COVID-19 to control its spread and ensure the well-being of individuals. This study investigated the impact of COVID-19 and societal lockdown measures on the mental health of adults in the UAE. A cross-sectional study was conducted using an English and Arabic online questionnaire between May and June 2020. The psychological impact was assessed by the Impact of Event Scale-Revised (IES-R), and the social and family support impact was evaluated using questions from the Perceived Support Scale (PSS). A total of 4,426 participants (3,325 females and 1,101 males) completed the questionnaire. The mean IES-R score was 28.0 ± 14.6, reflecting a mild stressful impact with 27.3% reporting severe psychological impact. Over 36% reported increased stress from work, home and financial matters. Also, 43–63% of the participants felt horrified, apprehensive or helpless due to COVID-19. Females, younger participants, part-timers, and college or University graduates were more likely to have a high IES-R score (*p* < 0.05). The majority of participants reported receiving increased support from family members, paying more attention to their mental health, and spending more time to rest and relax. The results of this study demonstrate the impact of the COVID-19 pandemic on mental health among the UAE residents and highlight the need to adopt culturally appropriate interventions for the general population and vulnerable groups, such as females and younger adults.

## Introduction

The novel coronavirus (COVID-19) pandemic has brought about extraordinary challenges in various aspects of life. It is highly expected that outbreaks lead to increase in unemployment and impaired financial status as well as compromised physical and mental health (1, 2). The novel coronavirus emerged initially in Wuhan, China in late December 2019 and surged exponentially across the world leading to the declaration of a global pandemic by the World Health Organization (WHO) on March 11, 2020 (3, 4). More than 105.4 million confirmed cases and over 2.3 million deaths were recorded globally as of 7 February 2021 (5). In the United Arab Emirates (UAE), the first cases of positive coronavirus were diagnosed on January 23, 2020; a Chinese family of four visiting the UAE on holiday (6). To date, there have been over 323 thousand confirmed cases and a total of 914 deaths in the UAE (5).

The alarming spread of the disease and its inevitable health and socioeconomic impact has led to the implementation of serious measures across the world. This was manifested by borders closure, suspension of flights, complete and partial lockdowns, quarantine, physical distancing, and mandating public respiratory hygiene measures (7). During the UAE countrywide lockdown, imposed between mid-March and July 31, 2020, people were instructed to stay at home other than for important individual movement (8). Moreover, the government closed non-essential business (e.g., cafes, gyms, theme parks, salons, and spas), initiated telework and distance learning, improved delivery services like delivering drugs to chronically ill patients and sanitized cities during the night as part of the national disinfection program (9). By the end of the lockdown on early July 2020, reopening of businesses and economic activities was initiated, but with strict preventative and restriction measures including overnight curfews, movement restrictions at the local level, physical distancing and wearing of face covering in public spaces (10).

Quarantine has been historically implemented to control the spread of infectious diseases outbreaks; however, it represents an unfavorable experience for the general population (11). Literature shows that multiple stressors including movement restriction, separation from family and friends, uncertain future, fear of infection, distress, loneliness, boredom, and financial loss are all factors that may exacerbate negative psychological impact and play a role in aggravating poor mental health (12, 13). Several studies have explored mental health problems (emotional disturbance, depression, fear of infection, stress, post-traumatic stress symptoms, and irritability) during other infectious and widespread outbreaks like the Severe Acute Respiratory Syndrome (SARS) epidemic in 2003 and the Middle East respiratory syndrome coronavirus (MERS-CoV) in 2012 (14, 15). However, MERS-CoV was not considered a pandemic because of the low rates of reported cases. Unlike SARS and MERS-CoV the psychological impact of the current pandemic might be more profound due to extensive social media exposure, increased global connectivity, high transmission rates and long duration of quarantine (16, 17).

There is limited research examining mental health in the UAE. However, available studies indicated high prevalence of depression and anxiety among primary health care attenders (18, 19) and social stigma was the main challenge associated with seeking and utilizing psychological services in the UAE (20). Considering the prevalence of mental health disorders in the UAE and in light of the current pandemic, the Ministry of Health and Prevention in the UAE launched a Hayat (life) program from mental health support during the COVID-19 pandemic and a dedicated telephone counseling hotline to help those with psychological concerns or anxiety (10). However, the impact of COVID-19 on mental health status of UAE population has not been investigated to-date.

The lockdown and quarantine during COVID-19 have resulted major social and psycho-logical impact on the whole population (21). The pandemic has caused changes on societal level as some families experienced conflicts, and instability due to the restriction measures implemented during the outbreak (22). However, many have considered this as an opportunity to establish better support and bonds between family members (23). In countries like the UAE, extended family is considered a pillar of the society and to no doubt have the ability to lessen the negative impacts of such health crisis. Nonetheless, limited research is available on how people are affected by the pandemic and the impact it has on their mental health and living conditions in the UAE. This study aimed to investigate the impact of the COVID-19 outbreak and lockdown measures on mental health and well-being among residents of the UAE. The pandemic is not over yet and restriction measures, teleworking, and home-schooling of children still apply in the UAE. Therefore, it was hypothesized that specific factors related to the implementation of restrictive measures may be associated with the inevitable increase in psychological distress among the general population.

## Materials and Methods

### Study Design, and Participants

A cross-sectional web-based research study was conducted from May 11, 2020 to June 15, 2020 in the UAE. A total of 4,426 participants were recruited from all the seven emirates in the UAE (Abu Dhabi, Dubai, Sharjah, Ajman, Um Al Quwain, Ras Al-Khaimah, and Fujairah). The study inclusion criteria were, living in the UAE and age ≥18 years. Participants were invited electronically to participate in the study using convenience and snowball sampling methods. These methods guarantee large-scale dissemination and recruitment of participants.

The Impact of Event Scale-Revised (IES-R) was used to assess the psychological impact of the pandemic and the Perceived Support Scale (PSS) was employed to assess the impact on social and family support (24–26). The questionnaire was prepared on Google document forms in English and Arabic, then pilot tested for clarity in a sample of 26 people prior to large-scale launching. Minimal adjustments to the wording were made to guarantee understandability. A uniform resource locator (URL) was retrieved for the survey and was distributed formally (using e-mail invitations) and informally (using social media platforms, e.g., LinkedIn™, Facebook™, and WhatsApp™). The questionnaire included an information sheet on the first page, and the participants were asked to consent before completing the questionnaire. They were free to exit the survey at any point without giving explanations, and no personal identification was requested to retain information confidentiality. Participants were given no incentives for participation in the study. The system of Google Forms only provides responses for questionnaires with 100% completion rate. The responses were downloaded as an Excel file and securely stored using a password protected “Cloud” database.

The present study followed the ethical code for web-based research (27, 28) and conforms to the principles embodied in the Declaration of Helsinki (29). The study protocol was approved by the Social Sciences Research Ethics Committee at United Arab Emirates University (ERS_2020_6115). An electronic informed consent was obtained from all participants.

### Survey Questionnaire

Socio-demographic characteristics were collected including age, gender, education level, employment status, marital status, and work or study setting.

#### The Impact of Event Scale-Revised (IES-R)

The scale was used to assess the psychological impact of COVID-19 among adults in the UAE (24). The IES-R is a self-administered questionnaire that includes 22 items and has been previously translated and validated in the English and Arabic languages (30–33). It has been also validated to investigate trauma-related stress symptoms related to the short- and long-term impact of the COVID-19 outbreak (34). Moreover, the IES-R has also been used to measure symptomatology experienced during the COVID-19 pandemic in Saudi Arabia, Egypt, Italy, and China (26, 35–38).

Participants were asked to rate the items based on how distressing the COVID-19 pandemic was for them. Items are rated on a 5-point Likert scale, ranging from 0 (not at all) to 4 (extremely); the response for each question was scored and generated a total score (ranging from 0 to 88). The total IES-R score was considered normal (from 0 to 23); indicative of mild (from 24 to 32); moderate (from 33 to 36); or severe (≥37) psychological impact (35). Three subscale scores were also calculated measuring intrusion (eight items), avoidance (eight items), and hyperarousal (six items) (25).

#### Indicators of Negative Mental Health Impact

This section contained six modified and validated questions regarding negative mental health impacts caused by the COVID-19 pandemic (25). Three questions asked if the participants felt horrified, apprehensive, or helpless due to the pandemic. The remaining three questions asked if the participants are experiencing increased stress from work, financial status, and staying at home during the pandemic. The response options were much increased, increased, same as before, decreased, and much decreased.

#### Impact on Social and Family Support

This section included modified and validated questions from the Perceived Support Scale (PSS) assessing the impact of the COVID-19 pandemic on the support received from family or friends (25, 35). Participants were asked about; support from friends, support from family members, sharing feelings with a family member, sharing feelings with others when in blue, and caring for family members' feelings. The response options were much increased, increased, same as before, decreased, and much decreased.

#### Mental Health-Related Lifestyle Changes

Participants were asked to rate the frequency of mental health related lifestyle changes that might have affected them during COVID-19 pandemic using modified and validated questions from the Mental Health Lifestyle Scale (MHLSS) (25). This section comprised of four items; attention to mental health, spending enough time to rest, relax, and exercise. The response options were much increased, increased, same as before, decreased, and much decreased.

### Statistical Analysis

Normality of data was tested using Kolmogorov-Smirnov test. Categorical variables were presented as frequencies and percentages and continuous variables were presented as mean ± standard deviation (SD). A Chi-square (χ^2^) test was used to determine the association between IES-R categories with categorical variables. Independent t-test was used to determine differences in IES-R, intrusion, avoidance, and hyperarousal scores between males and females. When significance was detected the effect size (Cohen's effect size, d) was calculated and reported as described previously (39). Moreover, generalized liner model was carried out to determine the confounding effects of sociodemographic factors, negative mental health impact factors, social and family support indicators, and lifestyle factors on continuous IES-R total score. Univariant general linear model with a cut-off value of *p* < 0.02 was used to select factors to be included in the final regression model. A p-value < 0.05 was considered to be statistically significant. All data were analyzed using Statistical Package for the Social Sciences (SPSS) version 26.0 (IBM, Chicago, IL, USA). The minimum sample size (*n* = 1,579) was calculated using G^*^power software, version 3.1.9.4 (HHU, Germany) to detect small effect size (0.02), with a power of 0.95, and alpha 0.05.

## Results

### Sociodemographic Characteristics

The percentage of participants that completed the survey in the Arabic and English languages was 85.0 and 15.0%, respectively. The sociodemographic characteristics of the study population are presented in Table 1. The female to male ratio was almost 3:1, with 24.9% males. The majority of surveyed individuals were aged 36–45 years (33.9%), were married (67.7%), had completed college or University degree of education (64.5%), full-time employed (63.2%), and were working or studying from home (56.2%).

**Table 1:** Sociodemographic characteristics of participants (*n* = 4,426) using Chi-square test.

**Variables**	***n* (%)**
**GENDER**	
Female	3,325 (75.1)
Male	1,101 (24.9)
**AGE (YEARS)**	
18–25	736 (16.6)
26–35	1,006 (22.7)
36–45	1,499 (33.9)
46–55	894 (20.2)
>55	291 (6.6)
**MARITAL STATUS**	
Married	2,998 (67.7)
Single	1,189 (26.9)
Divorced/Widowed	239 (5.4)
**EDUCATION LEVEL**	
High school	662 (15.0)
College/University	2,853 (64.5)
Higher qualification	911 (20.6)
**EMPLOYMENT STATUS**	
Employed (Full-time)	2,796 (63.2)
Employed (Part-time)	300 (6.8)
Unemployed	1,330 (30.0)
**WORKING/STUDYING FROM HOME**	
Yes	2,488 (56.2)
No	1,536 (34.7)
Not applicable	402 (9.1)

### Impact of Event Scale-Revised (IES-R) by Gender

The overall mean IES-R score was 28.0 ± 14.6 (range 0–84), reflecting a mild stressful impact of the COVID-19 pandemic on the surveyed participants (Table 2). For 41.7% of the participants, the IES-R score was in the normal range (0–23). Over 27% of the participants had a score reflecting severe psychological impact (≥37), with a higher mean IES-R score among females (28.6 ± 14.9) compared to males (25.9 ± 13.7) (*p* < 0.001; with a small Cohen's effect size).

**Table 2:** Psychological impact of COVID-19 on participants by gender (*n* = 4,426).

**Variables**	**All (*n* = 4,426)**	**Females (*n* = 3,325)**	**Males (*n* = 1,101)**	*P*-value^*^	d^**^
**IES-R**	**Mean** **±** **SD**				
Total score	28.0 ± 14.6	28.6 ± 14.9	25.9 ± 13.7	<0.001	0.19
Intrusion	8.9 ± 5.9	9.0 ± 6.0	8.4 ± 5.6	0.002	0.11
Avoidance	11.8 ± 5.8	12.0 ± 5.9	11.3 ± 5.6	0.001	0.11
Hyperarousal	7.3 ± 5.0	7.6 ± 5.1	6.2 ± 4.8	<0.001	0.28

*IES-R, Impact of Event Scale–Revised; SD, Standard Deviation*,

* *p-value was based on independent t-test*;

** *Cohen's effect size*.

The overall means for intrusion, avoidance and hyperarousal scales in participants were 8.9 ± 5.9, 11.8 ± 5.8, and 7.3 ± 5.0, respectively. The mean scores for all subscales in females were significantly higher compared with males (*p* < 0.001; with a small Cohen's effect size).

### Sociodemographic and Impact Event Scale-Revised (IES-R)

Table 3 presented the association of IES-R scores with sociodemographic factors. A Chi-square analysis revealed significant association between IES-R categories with gender (*p* < 0.001), age (*p* < 0.001), education level (*p* = 0.002), and employment status (*p* = 0.02). Multivariate regression analysis revealed that females (*p* < 0.001), younger participants (age groups 18–25, 26–35, and 36–45; *p* < 0.001), college/University graduate (*p* = 0.004), and part-timers (*p* = 0.033) were more likely to have higher IES-R scores.

**Table 3:** Association of IES-R scores with sociodemographic factors (*n* = 4,426).

**Variables**	**All *n* = 4,424**	**IES-R categories**				*P*-value^*^	**Rate ratio (CI 95%)**	*P*-value^**^
		**Normal *n* (%) *n* = 1,846**	**Mild *n* (%) *n* = 1,002**	**Moderate *n* (%) *n* = 371**	**Severe *n* (%) *n* = 1,207**			
**GENDER**								
Female	3,325 (75.1)	1,323 (71.7)	740 (73.9)	289 (77.9)	973 (80.6)	<0.001	10.143 (3.547–29.002)	<0.001
Male	1,101 (24.9)	523 (28.3)	262 (26.1)	82 (22.1)	234 (19.4)		1	
**AGE (YEARS)**								
18–25	736 (16.6)	313 (17.0)	140 (14.0)	67 (18.1)	216 (17.9)	<0.001	11.374 (1.407–91.971)	<0.001
26–35	1,006 (22.7)	374 (20.3)	234 (23.4)	86 (23.2)	312 (25.8)		73.036 (10.426–511.637)	
36–45	1,499 (33.9)	609 (33.0)	329 (32.8)	122 (32.9)	439 (36.4)		19.309 (2.956–126.137)	
46–55	894 (20.2)	409 (22.2)	216 (21.6)	74 (19.9)	195 (16.2)		1.441 (0.206–10.088)	
>55	291 (6.6)	141 (7.6)	83 (8.3)	22 (5.9)	45 (3.7)		1	
**MARITAL STATUS**								
Married	2,998 (67.7)	1,258 (68.1)	701 (70.0)	252 (67.9)	787 (65.2)	0.281		
Single	1,189 (26.9)	491 (26.6)	256 (25.5)	96 (25.9)	346 (28.7)			
Divorced/Widowed	239 (5.4)	97 (5.3)	45 (4.5)	23 (6.2)	74 (6.1)			
**EDUCATION LEVEL**								
High school	662 (15.0)	286 (15.5)	166 (16.6)	42 (11.3)	168 (13.9)	0.002	0.592 (0.128–2.729)	0.004
College/University	2,853 (64.5)	1,154 (62.5)	617 (61.6)	266 (71.7)	816 (67.6)		3.493 (1.142–10.683)	
Higher degree	911 (20.6)	406 (22.0)	219 (21.9)	63 (17.0)	223 (18.5)		1	
**EMPLOYMENT STATUS**								
Full-time	2,796 (63.2)	1,172 (63.5)	599 (59.8)	234 (63.1)	791 (65.5)	0.020	3.103 (1.132–8.506)	0.033
Part-time	300 (6.8)	110 (6.0)	78 (7.8)	20 (5.4)	92 (7.6)		7.404 (1.173–46.729)	
Unemployed	1,330 (30.0)	564 (30.6)	325 (32.4)	117 (31.5)	324 (26.8)		1	
**WORKING FROM HOME**								
Yes	2,488 (56.2)	1,014 (54.9)	541 (54.0)	222 (59.8)	711 (58.9)	0.060		
No	1,536 (34.7)	654 (35.4)	376 (37.5)	122 (32.9)	384 (31.8)			
Not applicable	402 (9.1)	178 (9.6)	85 (8.5)	27 (7.3)	112 (9.3)			

*IES-R, Impact of Event Scale–Revised; CI, confidence interval*;

* *p-value was based on Chi-square test*;

** * p-value was based on generalized linear model analysis*.

### Negative Mental Health Indicators and Impact Event Scale-Revised (IES-R)

Association of IES-R categories with negative mental health indicators are displayed in Table 4. Over 43% of the participants reported increased stress from work during the outbreak, 36.5% felt an increased level of stress from financial matters, and 55.7% of the participants reported increased stress at home during the pandemic. Moreover, around 43–63% of participants felt horrified, apprehensive or helpless due to the pandemic. Chi-square analysis and multivariate regression analysis both revealed that increased stress and negative feelings were strongly associated with higher IES-R scores (*p* <0.001).

**Table 4:** Association of IES-R scores with negative mental health indicators (*n* = 4,426).

**Variables**	**All *n* = 4,424**	**IES-R categories**				*P*-value^*^	**Rate ratio (CI 95%)**	*P*-value^**^
		**Normal *n* (%) *n* = 1,846**	**Mild *n* (%) *n* = 1,002**	**Moderate *n* (%) *n* = 371**	**Severe *n* (%) *n* = 1,207**			
**INCREASED STRESS FROM WORK**								
No	2,505 (56.6)	1,270 (68.8)	581 (58.0)	192 (51.8)	462 (38.3)	<0.001	1	<0.001
Yes	1,921 (43.4)	576 (31.2)	421 (42.0)	179 (48.2)	745 (61.7)		34.274 (15.642–75.096)	
**INCREASED FINANCIAL STRESS**								
No	2,812 (63.5)	1,368 (74.1)	648 (64.7)	225 (60.6)	571 (47.3)	<0.001	1	<0.001
Yes	1,614 (36.5)	478 (25.9)	354 (35.3)	146 (39.4)	636 (52.7)		11.842 (5.254–26.694)	
**INCREASED STRESS FROM HOME**								
No	1,961 (44.3)	1,115 (60.4)	433 (43.2)	124 (33.4)	289 (23.9)	<0.001	1	< <0.001
Yes	2,465 (55.7)	731 (39.6)	569 (56.8)	247 (66.6)	918 (76.1)		37.007 (16.004–85.575)	
**FELT HORRIFIED DUE TO COVID-19**								
No	1,620 (36.6)	1,034 (56.0)	339 (33.8)	91 (34.5)	156 (12.9)	<0.001	1	<0.001
Yes	2,806 (63.4)	812 (44.0)	663 (66.2)	280 (75.5)	1,051 (87.1)		444.959 (168.382–1175.829)	
**FELT APPREHENSIVE DUE TO COVID-19**								
No	1,755 (39.7)	1,090 (59.0)	366 (36.5)	93 (25.1)	206 (17.1)	<0.001	1	<0.001
Yes	2,671 (60.3)	756 (41.0)	636 (63.5)	278 (74.9)	1,001 (82.9)		25.755 (9.693–68.433)	
**FELT HELPLESS DUE TO COVID-19**								
No	2,518 (56.9)	1,361 (73.7)	599 (59.8)	176 (47.4)	382 (31.6)	<0.001	1	<0.001
Yes	1,908 (43.1)	485 (26.3)	403 (40.2)	195 (52.6)	825 (68.4)		204.424 (88.301–473.258)	

*Answers of “much increased” and “increased” have been merged as “Yes”; Answers of “same as before”, “decreased” and “much decreased” have been merged as “No”; IES-R, Impact of Event Scale–Revised; CI, confidence interval*;

* *p-value was based on Chi-square test*;

** * p-value was based on generalized linear model analysis*.

### Impact on Social and Family Support

As expected, Table 5 showed that 45.1% of the participants reported receiving increased support from family members, 52.8% reported increased shared feelings with their family members and 71.8% cared more about their family members' feelings during the pandemic. In contrast, only 27% had increased support from friends. However, participants with increased support from family and friends, who shared feelings with family members, and caring about family members were more likely to report higher IES-R scores (*P* <0.001).

**Table 5:** Association of IES-R scores with impact on family and social support (*n* = 4,424).

**Variables**	**All**	**IES-R categories**				*P*-value^*^	**Rate ratio (CI 95%)**	*P*-value^**^
		**Normal *n* (%) *n* = 1,846**	**Mild *n* (%) *n* = 1,002**	**Moderate *n* (%) *n* = 371**	**Severe *n* (%) *n* = 1,207**			
**GETTING SUPPORT FROM FRIENDS**								
Decreased	811 (18.3)	335 (18.1)	173 (17.3)	64 (17.3)	239 (19.8)	<0.001	1	<0.001
Same as before	2,418 (54.6)	1,169 (63.3)	534 (53.3)	188 (50.7)	527 (43.7)		0.539 (0.145–2.008)	
Increased	197 (27.0)	342 (18.5)	295 (29.4)	119 (32.1)	441 (36.5)		5.902 (1.336–26.076)	
**GETTING SUPPORT FROM FAMILY MEMBERS**								
Decreased	430 (9.7)	177 (9.6)	79 (7.9)	39 (10.5)	135 (11.2)	<0.001	1	0.016
Same as before	1,999 (45.2)	1,026 (55.6)	417 (41.6)	139 (37.5)	417 (34.5)		1.087 (0.180–6.545)	
Increased	1,997 (45.1)	643 (34.8)	506 (50.5)	193 (52.0)	655 (54.3)		5.042 (0.793–32.073)	
**SHARED FEELINGS WITH FAMILY MEMBERS**								
Decreased	21 (11.8)	194 (10.5)	99 (9.9)	55 (14.8)	173 (14.3)	<0.001	1	<0.001
Same as before	1,566 (35.4)	894 (48.4)	318 (31.7)	87 (23.5)	267 (22.1)		0.026 (0.005–0.135)	
Increased	2,339 (52.8)	758 (41.1)	585 (58.4)	229 (61.7)	767 (63.5)		0.501 (0.096–2.610)	
**SHARED FEELINGS WITH OTHER WHEN IN BLUE**								
Decreased	45 (21.4)	344 (18.6)	222 (22.2)	94 (25.3)	285 (23.6)	<0.001	1	<0.001
Same as before	2,181 (49.3)	1,144 (62.0)	495 (49.4)	146 (39.4)	396 (32.8)		0.028 (0.008–0.096)	
Increased	1,300 (29.4)	358 (19.4)	285 (28.4)	131 (35.3)	526 (43.6)		4.933 (1.306–18.624)	
**CARING FOR FAMILY MEMBERS' FEELINGS**								
Decreased	75 (4.0)	65 (3.5)	37 (3.7)	14 (3.8)	59 (4.9)	<0.001	1	<0.001
Same as before	1,071 (24.2)	652 (35.3)	194 (19.4)	57 (15.4)	168 (13.9)		0.054 (0.005–0.586)	
Increased	3,180 (71.8)	1,129 (61.2)	771 (76.9)	300 (80.9)	980 (81.2)		2.753 (0.284–26.660)	

*Answers of “much increased” and “increased” have been merged; Answers of “decreased” and “much decreased” have been merged; IES-R, Impact of Event Scale–Revised; CI, confidence interval*;

* *p-value was based on Chi-square test*;

** * p-value was based on generalized linear model analysis*.

### Mental Health-Related Lifestyle Changes

Table 6 showed the association of IES-R scores with lifestyle indicators during the pandamic. A significat percentage of participants reported increased attention to their mental health (45.5%) and spending more time to rest and relax (41.2 and 38.7%, respectively). In contrast, 41.0% of the participants reported spending less time exercising. The multivariate regression analysis showed that participants who had increased attention to mental health (*p* <0.001) and decreased time spent on resting (*p* = 0.002), relaxing (*p* <0.001), and exersicing (*p* < 0.001), were more likely to report higher IES-R scores compared with particpants reporting no change.

**Table 6:** Association of IES-R scores with lifestyle changes (*n* = 4,424).

**Variables**	**All**	**IES-R categories**				*P*-value^*^	**Rate ratio (CI 95%)**	*P*-value^**^
		**Normal *n* (%) *n* = 1,846**	**Mild *n* (%) *n* = 1,002**	**Moderate *n* (%) *n* = 371**	**Severe *n* (%) *n* = 1,207**			
**PAY ATTENTION TO MENTAL HEALTH**								
Decreased	283 (6.4)	92 (5.0)	58 (5.8)	22 (5.9)	111 (9.2)	<0.001	1	<0.001
Same as before	2,130 (48.1)	1,088 (58.9)	456 (45.5)	167 (45.0)	419 (34.7)		0.044 (0.007–0.264)	
Increased	2,013 (45.5)	666 (36.1)	488 (48.7)	182 (49.1)	677 (56.1)		15.164 (2.512–91.553)	
**TIME SPENT TO REST**								
Decreased	939 (21.2)	262 (14.2)	192 (19.2)	100 (27.0)	385 (31.9)	<0.001	1	0.002
Same as before	1,662 (37.6)	845 (45.8)	356 (35.5)	101 (27.2)	360 (29.8)		0.048 (0.009–0.263)	
Increased	1,825 (41.2)	739 (40.0)	454 (45.3)	170 (45.8)	462 (38.3)		0.111 (0.017–0.723)	
**TIME SPENT TO RELAX**								
Decreased	1,056 (23.9)	289 (15.7)	215 (21.5)	112 (30.2)	440 (36.5)	<0.001	1	<0.001
Same as before	1,658 (37.5)	858 (46.5)	361 (36.0)	109 (29.4)	330 (27.3)		0.008 (0.002–0.043)	
Increased	1,712 (38.7)	699 (37.9)	426 (42.5)	150 (40.4)	437 (36.2)		0.025 (0.004–0.155)	
**TIME SPENT TO EXERCISE**								
Decreased	1,816 (41.0)	631 (34.2)	446 (44.5)	159 (42.9)	580 (48.1)	<0.001	1	<0.001
Same as before	1,492 (33.7)	757 (41.0)	312 (31.1)	116 (31.3)	307 (25.4)		0.062 (0.022–0.169)	
Increased	1,118 (25.3)	458 (24.8)	244 (24.4)	96 (25.9)	320 (26.5)		0.258 (0.087–0.767)	

*Answers of “much increased” and “increased” have been merged; Answers of “decreased” and “much decreased” have been merged; IES-R, Impact of Event Scale–Revised; CI, confidence interval*;

* *p-value was based on Chi-square test*;

** * p-value was based on generalized linear model analysis*.

## Discussion

The results of this study showed that over one third of the participants in the UAE had an IES-R score indicating moderate to severe disturbance due to the COVID-19 pandemic. Similarly, in neighboring gulf countries, an online survey conducted among Saudi adults during the pandemic reported mild to moderate rates of anxiety among the general population and a significantly higher level of anxiety was observed among married respondents (40). In Bahrain, an online Depression Anxiety and Stress Scale-21 (DASS-21) showed that one third of the participants had depressive and stress symptoms (41). Likewise, Lebanese citizens have also reported an increase of Post-traumatic Stress Disorder (PTSD) symptomatology during the fourth week of the COVID-19 quarantine (42). However, levels in the current study were lower than those reported in China, which revealed that over half (53.8%) of the general population had a moderate-to-severe psychological impact during the outbreak (13). Similar to China, results from Egypt indicated high IES-S mean score (34.3 ± 15.0), and more than half of the participants (52%) showed moderate and severe psychological impact due to the pandemic (38). Different populations in the world have been experiencing different pandemic fear depending on the speed of spreading, regulations adopted by the governments or previous experiences of outbreaks like those caused by SARS, Ebola, and MERS-CoV, such factors could contribute to heightening the impact of the present pandemic (43). The majority of the participants in the current study reported feeling horrified, apprehensive or helpless due to the pandemic. Existing evidence suggests a link between hopelessness and depression and highlights the unique sensory processing patterns of depressed individuals in determining unfavorable outcomes (44).

In the current study, females, younger participants, part-timers and University or college educated participants were more likely to have higher stress scores. The process underlying gender differences in the susceptibility to psychological disorders have not yet been fully understood. However, some evidence suggests that fluctuations in ovarian hormone levels and greater brainstem activation among women may contribute to greater PTSD prevalence and higher emotional stimuli (45–47). Moreover, the literature suggested that greater access to information through social media could be triggering stress and anxiety amongst the younger population (48, 49). Conflicting results about the potential relationship between education level and PTSD were reported in the literature. Some evidence suggests that individuals with a higher level of education might use better coping strategies and ultimately be less impacted by the environmental disaster (50, 51). Others suggested that highly educated people might be more stressed due to higher self-awareness and discernment of the pandemic severity (48, 52).

Findings of the current study were in agreement with results from Saudi Arabia, as health care workers, students and females had higher levels of stress, anxiety and depression symptoms (37). Likewise, females, younger persons, students, those with chronic illnesses and people with low income reported higher psychological impacts due to COVID-19 in Egypt and Bahrain (38, 41). Findings from Tunisia identified females, people who reported exposure to confirmed COVID-19 case, those who felt deprived of essential resources, and those exposed to 2 or more hours per day of media coverage of COVID-19 as vulnerable groups (53). These subgroups are considered at higher risk for adverse psychological effects during such crisis. Therefore, clinical interventions targeted toward vulnerable groups are needed to mitigate the influence of the ongoing pandemic and alleviate triggers of distress, such as low social, financial, and emotional support, feelings of fear, isolation and uncertainty, and threats to health and well-being (54). Telehealth counseling helplines have been shown useful to provide support to the vulnerable groups and appropriate for the delivery of mental health services (55). Likewise, awareness about self-relaxation and self-care measures can lessen feelings of social isolation (56).

The study showed that over one-third of participants experienced increased stress from work, home and financial matters during the COVID-19 pandemic. The results were comparable to reports among Egyptian adults (38). Contributing factors may include effects of COVID-19 and the associated lockdowns on daily life and routine, work-family balance, and lack of financial support for those who need it (57). Moreover, COVID-19 associated lockdowns required many working parents to do a full-time job from home and care for the family simultaneously. Findings also suggested that females were more likely to have experienced an increased level of stress from work while males experienced an increased level of stress from financial matters. Considering the long-standing role expectations of females as caregivers and males as breadwinners, it is usually working mothers, who need to adjust their work patterns to meet the needs of the family and the household (58). A study among Australian working parents revealed that active care and household management rose by an hour and a half for fathers and by 2 h and a half for mothers (57). Demonstrating that both genders were dissatisfied with their work-family balance and facing increased stress from home matters during the COVID-19 pandemic, which was also shown in the current study. Besides, families were affected by prolonged school closure, requiring online education support and uncertainty about examinations and enrolment arrangements (59). Governments and workplace policies could support work-family balance by allowing the right to request part-time work, flexible working hours, and the option to work from home (60).

The majority of participants reported getting increased support from other family members as well as caring more about the feelings of family as a whole during the pandemic. Apparently, such acts have had a positive impact on mental health and may have helped the participants to cope with other negative feelings during the pandemic. Similarly, a study from Egypt confirmed that family and friends were much valued in a time of crisis (38). On the other hand, domestic violence reports have increased during the pandemic in many parts of the world. The World Health Organization Europe member states have reported a 60% increase in emergency calls from women subjected to violence by their intimate partner during the pandemic (61). Reasons could include job losses, rising alcohol-based harm and drug use, stress and fear (61).

Current results revealed strong association between decreased time spent on physical activity and likelihood of scoring higher on IES-R scale, suggesting that lower levels of physical activity during the pandemic are more likely to increase impact of the event in a negative manner. These results are in agreement with the results reported by a study among Arab adults that investigated the influence of home confinement during the pandemic and reported significant relationship between higher levels of physical activity and better mental well-being (62). The authors of the latter study suggested that higher levels of physical activity are associated with positive hormonal status, therefore, favoring improved mood and mental health (62). Moreover, physical activity has been recommended as a form of therapy to counteract the expected negative impact of quarantine on mental and physical factors (63). The authors of the current study suggest that home-based physical activities could be employed to overcome the closure of training facilities and public parks during lockdown to improve mental status.

This study has several strengths, including the large sample size and the use of validated questionnaires that provide the ability to compare the findings with previous studies. Moreover, due to the strict quarantine measures in place, using an online survey allowed data collection from various cities and guaranteed the anonymity of the participants. However, there were some limitations; the use of a self-reported questionnaire which might cause some respondent bias or misreporting of data. Also, the snowballing sampling strategy which may limit the representativeness of the UAE population. Furthermore, the use of an online survey limited the reach to non-social media users which led to less generalizable results. The cross-sectional study design may limit the causal interpretation, and a longitudinal study on the psychological impact in the UAE is recommended.

## Data Availability Statement

The raw data supporting the conclusions of this article will be made available by the authors, without undue reservation.

## Ethics Statement

The study protocol was reviewed and approved by the Social Sciences Research Ethics Committee at United Arab Emirates University (ERS_2020_6115). An electronic informed consent was obtained from all participants.

## Author Contributions

ASA, MB, and LC: conceptualization. ASA, MB, MM, AA, LS, MH, SS, and LC: methodology. ASA, MM, SS, and LC: formal analysis and writing—original draft preparation. ASA, MB, MM, AA, AAM, AJ, DA, HIA, HA, HH, LS, MH, RS, SS, TO, and LC: investigation and writing—review and editing. All authors have read and agreed to the published version of the manuscript.

## Conflict of Interest

The authors declare that the research was conducted in the absence of any commercial or financial relationships that could be construed as a potential conflict of interest.
